# Large T antigen mediated target gene replication improves site-specific recombination efficiency

**DOI:** 10.3389/fbioe.2024.1377167

**Published:** 2024-04-26

**Authors:** Zening Wang, Chuan Chen, Xin Ge

**Affiliations:** ^1^ Institute of Molecular Medicine, University of Texas Health Science Center at Houston, Houston, TX, United States; ^2^ Department of Chemical and Environmental Engineering, University of California Riverside, Riverside, CA, United States

**Keywords:** site-specific recombinase, RMCE, genome editing, large T antigen, directed evolution

## Abstract

With advantages of high-fidelity, monoclonality and large cargo capacity, site-specific recombination (SSR) holds great promises for precise genomic modifications. However, broad applications of SSR have been hurdled by low integration efficiency, and the amount of donor DNA available in nucleus for SSR presents as a limiting factor. Inspired by the DNA replication mechanisms observed in double-stranded DNA virus SV40, we hypothesized that expression of SV40 large T antigen (TAg) can increase the copy number of the donor plasmid bearing an SV40 origin, and in consequence promote recombination events. This hypothesis was tested with dual recombinase-mediated cassette exchange (RMCE) in suspension 293F cells. Results showed that TAg co-transfection significantly enhanced SSR in polyclonal cells. In the monoclonal cell line carrying a single landing pad at an identified genomic locus, 12% RMCE efficiency was achieved, and such improvement was indeed correlated with donor plasmid amplification. The developed TAg facilitated RMCE (T-RMCE) was exploited for the construction of large libraries of >10^7^ diversity, from which GFP variants with enhanced fluorescence were isolated. We expect the underlying principle of target gene amplification can be applicable to other SSR processes and gene editing approaches in general for directed evolution and large-scale genomic screening in mammalian cells.

## Introduction

Site-specific recombination (SSR) is a DNA rearrangement process in which exchange of strands occurs between two defined short sequences termed recognition sites (O’Gorman et al., 1991). This process involves recombinase-catalyzed breaking and rejoining of DNA strands without DNA synthesis, degradation, or the aid of enzyme cofactors (Grindley et al., 2006). Self-sufficiency together with the distinct and strict sequence specificities exhibited by most SSRs assures high-fidelity of DNA modifications (Inniss et al., 2017; Hamaker and Lee, 2018). Another advantage of SSR is its large cargo capacity—integration of segments >100 kb has been reported (Wallace et al., 2007). Possessing these features, SSR-mediated genetic manipulations have been broadly applied both *in vitro* and *in vivo*, ranging from generation of mini circle DNA and cell line development to stem cell programing and establishments of transgenic animals (Kay et al., 2010; Hamaker and Lee, 2018; Turan et al., 2013; Osterwalder et al., 2010; Tian and Zhou, 2021). Besides these long-standing uses, SSR has also been employed for the construction of combinatorial libraries, for applications such as affinity maturation of bi-specific antibodies and TCRs (Dilchert et al., 2022; Segaliny et al., 2023), Fc engineering (Chen et al., 2023) and large parallel screening of CRISPR gRNA libraries (Xiong et al., 2021). While remarkably accurate and versatile, one notable limitation of SSR is its relatively low efficiency—typical occurrence is less than 1% among transfected cells. To improve SSR efficiency, considerable efforts have been made, such as recombinase screening (Durrant et al., 2023) and engineering (Buchholz et al., 1998; Bolusani et al., 2006; Gaj et al., 2014; Voziyanova et al., 2016), and optimizations of recognition sites (Shah et al., 2015), recombinase expression cassette (Anderson et al., 2012) and transfection DNA amount and ratio (Voziyanova et al., 2013). So far, libraries of 10^4^–10^5^ diversities have been readily constructed via SSR (Matreyek et al., 2017; Xiong et al., 2021; Dilchert et al., 2022), but large diversity, *i.e.*, 10^7^ or more, which is often needed in directed evolution tasks, has not been fully achieved.

To allow SSR-mediated genomic integration to occur, donor DNA that encodes the transgene, once introduced into cytosol, must reach the recognition site pre-inserted on the chromosome (the landing pad). However, this trafficking through the cytoplasmic space and ultimate translocation across the nuclear envelope presents a significant barrier to gene delivery (Rosazza et al., 2013; Bai et al., 2017). In fact, following lipofection, only a small fraction of cytoplasmic plasmids can enter the nucleus (Coonrod et al., 1997; Tseng et al., 1997). Thus, one can reason that by increasing the amount of donor DNA available in nucleus, the SSR efficiency can be improved. To facilitate nuclear translocation of DNA, numerous approaches have been developed, *e.g.*, by incorporating transcription factor binding sites (Ondrej et al., 2007; Badding et al., 2012) or DNA nuclear targeting sequences into the transgene plasmids (Graessmann et al., 1989), or conjugating donor DNA with nuclear localization signals (NLS) (Hebert, 2003; Escriou et al., 2003). Apart from enhancing DNA delivery, in this study, we aim to test an alternative and conceptually novel strategy, *i.e.*, by amplification of donor plasmid DNA within nucleus.

**FIGURE 1:**
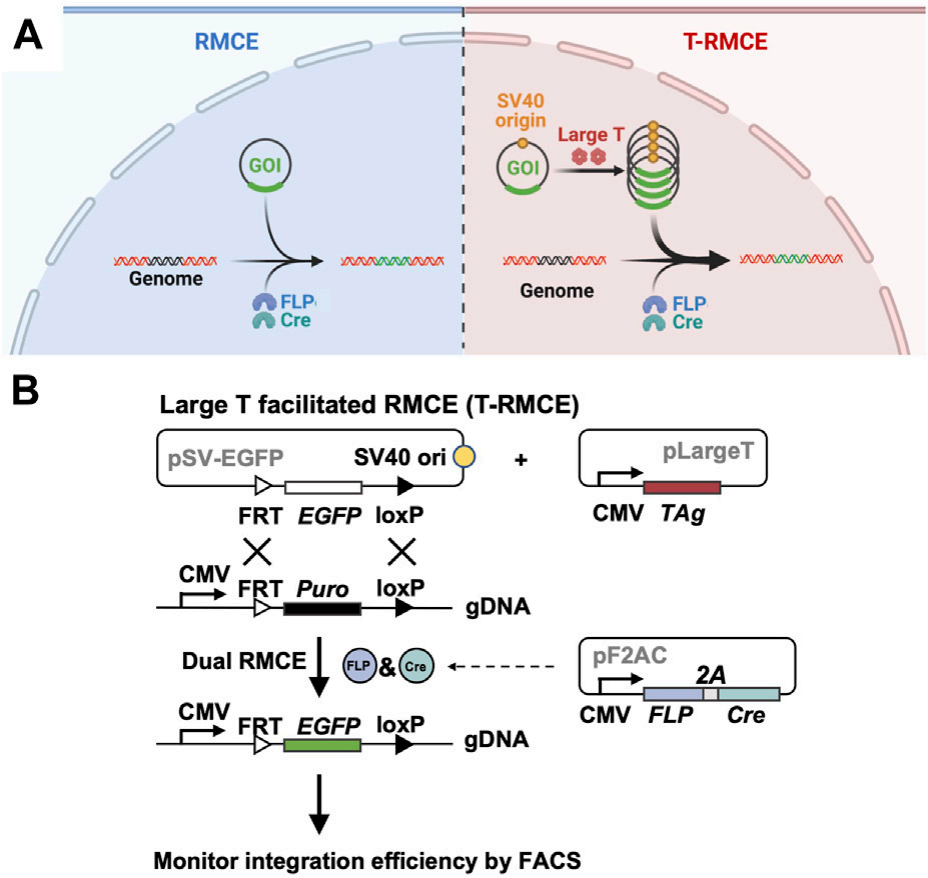
Large T facilitated RMCE (T-RMCE). **(A)** Proposed mechanism. In conventional RMCE (left), the donor plasmid carrying the gene of interest (GOI) was integrated to the landing pad on genome by recombinases FLP and Cre. Due to transportation barriers, only limited copies of GOI can reach to nucleus, and results in low integration efficiency. In T-RMCE (right), large T antigen mediates replication of the donor plasmid that bears a SV40 origin, thereby providing more copies of GOI available for integration and leading to improved RMCE efficiency. **(B)** Gene constructions and design principle. RMCE-competent cells are co-transfected with three plasmids: a SV40 origin containing promoter-less donor plasmid pSV-EGFP, a large T antigen expression plasmid pLargeT, and a plasmid encoding an expression cassette of FLP and Cre recombinases pF2AC. Expression of GOI, EGFP in this case, is enabled only after its integration between the recognition sites located downstream of a CMV promotor in the landing pad, and thus the (T-)RMCE efficiency can be measured by monitoring the percentages of EGFP^+^ cells.

Double-stranded DNA viruses manage to replicate their genomes at high efficiency. Particularly, polyomaviruses are capable of infecting growth-arrested differentiated cells, indicating a robust DNA amplification mechanism. As the most extensively studied member of the *Polymaviridae* family, simian virus 40 (SV40) carries a circular 5.2 kb dsDNA genome, encoding three structural virion proteins and two nonstructural proteins called small T antigen (tAg) and large T antigen (TAg) (Fanning and Zhao, 2009). The 708 aa multi-domain TAg belongs to the early coding unit of SV40, and performs important functions including viral DNA replication, virion assembly, and transcriptional regulation (Sullivan and Pipas, 2002; An et al., 2012). A series of structural biology studies has collectively elucidated TAg’s mode of action on SV40 origin distortion and DNA unwinding (Li et al., 2003; Gomez-Lorenzo et al., 2003; Gai et al., 2004; Meinke et al., 2007; Cuesta et al., 2010)—first, TAg monomers assemble at the replication origin to form a double hexamer in a head-to-head orientation; then with energy provided by the ATPase domain, TAg’s helicase activity unwinds dsDNA bidirectionally; and finally host’s replication machineries (*e.g.*, ssDNA binding protein, α-primase, topoisomerase I, and DNA polymerase) are recruited to accomplish the viral DNA amplification (An et al., 2012). Intriguingly, TAg is the only viral component needed to direct SV40 genome replication, a process that has been successfully reconstituted *in vitro* (Waga and Stillman, 1994). Furthermore, in the presence of SV40 TAg, transfected plasmids bearing an SV40 origin can be replicated in cells and maintained at a high copy number (Mahon, 2011). Therefore, we hypothesize that SV40 TAg mediated donor plasmid DNA amplification can improve SSR efficiency (Figure 1A). In this study, we test our hypothesis with dual recombinase-mediated cassette exchange (RMCE) (Lauth et al., 2002), in which two tyrosine recombinases are used to achieve precise genomic integration—we thus name the developed TAg facilitated RMCE method as T-RMCE. However, the underlying principle proved here should be applicable to other SSR processes (*e.g.*, using single or serine recombinases) and gene editing approaches in general (*e.g.*, using programable nucleases), where donor DNA availability may present as a limiting factor.

## Results

### Design of TAg facilitated RMCE (T-RMCE)

To test the hypothesis that TAg-mediated transgene replication can improve genomic integration efficiency of RMCE, three plasmids were designed for co-transfection (Figure 1B) two expression plasmids for TAg and a pair of SSR FLP and Cre respectively, and Cre, and a promoter-less donor plasmid encoding GOI (*e.g.*, EGFP) flanked by the associated SSR recognition sites FRT and loxP (exchange cassette FRT-EGFP-loxP). Importantly, the 130 bp SV40 origin sequence was cloned into the donor plasmid allowing TAg-mediated DNA replication. RMCE competent cells were prepared by genomic integration of a landing pad, which is composed of a constitutive promoter (*e.g.*, CMV) followed by a resistance marker gene (*e.g.*, Puro) located between FRT and loxP sites (cassette FRT-Puro-loxP). It is expected that upon transfection of these three plasmids into cells bearing the landing pad, TAg will mediate the donor plasmid amplification and FLP and Cre will catalyze the gene exchange taking place between the Puro cassette on genome and the EGFP cassette provided by the donor plasmid. Notably, as the promoter on landing pad is located outside of the recombination sites, the GOI introduced by the promoter-less donor plasmid can only be expressed after genomic integration. Therefore, the RMCE efficiency can be conveniently measured by monitoring GOI expression.

### SV40 TAg co-transfection improved RMCE efficiency in polyclonal cells

**FIGURE 2:**
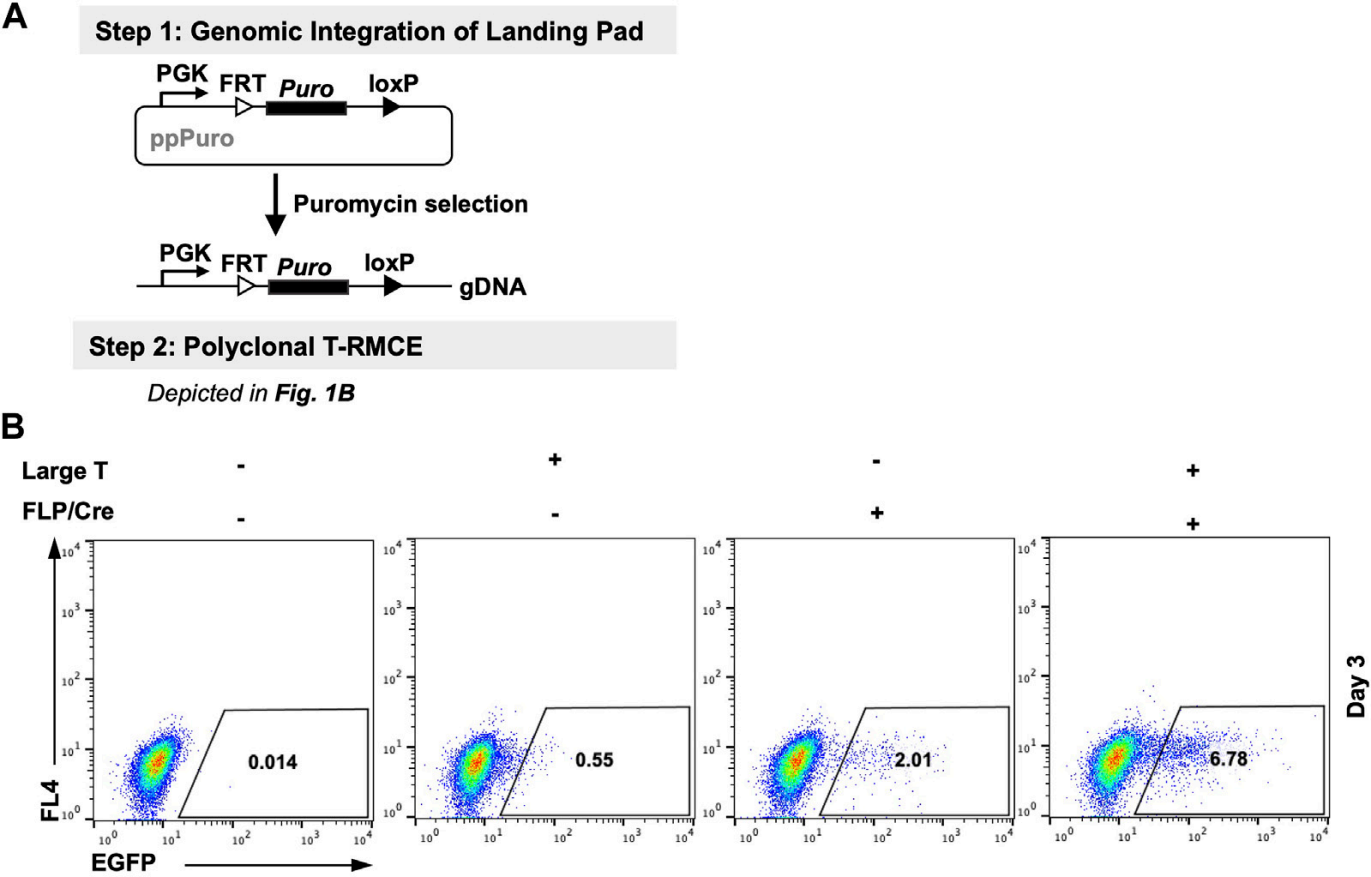
Large T antigen improved RMCE efficiency in polyclonal cells. **(A)** Experiment workflow. (Step 1) RMCE-competent polyclonal cells were generated by genomic integration of the landing pad carrying a PGK promoter and a puromycin resistance gene flanked by FRT and loxP recognition sites. (Step 2) Generated 293F-Puro polyclonal cells were subjected to T-RMCE as depicted in Figure 1B. **(B)** Flow cytometry results on day 3 after transfection with donor plasmid pSV-EGFP in the presence or absence of large T and/or FLP/Cre expression plasmids.

We first tested T-RMCE in polyclonal 293F cells in which the landing pad was stably integrated. More specifically, 293F cells were transfected with a landing pad plasmid encoding a PGK promoter and a downstream FRT-Puro-loxP cassette, and the stable cells were selected with puromycin (Figure 2A). The generated RMCE-competent 293F polyclonal cells were co-transfected with TAg and FLP-Cre expression and FRT-EGFP-loxP donor plasmids, and the T-RMCE efficiencies were evaluated by monitoring EGFP^+^ cell populations using flow cytometry. Results indicated that 6.8% of transfected cells were EGFP^+^ on day 3 post-transfection (Figure 2B), representing >3-fold improvement of integration efficiencies compared to regular RMCE at 2.0% without TAg.

### Generation of RMCE-competent 293F cells carrying single landing pads

Monoclonality (one variant per cell) is a highly desired feature for applications of combinatory libraries. To generate cells carrying single landing pads, we performed two sequential rounds of RMCE, a valid method described previously (Qiao et al., 2009; Chen et al., 2016) (Supplementary Figure S1). First, 293F cells were transfected with the landing pad plasmid carrying a CMV promoter, EGFP cassette (FRT-EGFP-loxP) and a hygromycin resistance gene. After selection with hygromycin, flow cytometry analysis showed that ∼38% of the survived cells were EGFP^+^, indicating that landing pad integration was achieved (Supplementary Figure S1, Step 1). To select the cell lines with high expression levels, top 10% EGFP^+^ cells were sorted out. At this stage, it is possible, though rare, that some cells may carry more than one landing pad in their genomes. To exclude these cells with multiple landing pads, isolated EGFP^+^ cells were cultured and subjected to RMCE1, in which promoter-less FRT-iRFP-loxP cassette was used as the donor DNA (Supplementary Figure S1, Step 2). On day 10 post-transfection, 0.04% of the cells exhibited iRFP^+^/EGFP^−^ signals, suggesting the occurrence of RMCE1. This population was isolated by FACS, and after expansion flow cytometry analysis confirmed that over 96% of the isolated cells were iRFP^+^ and EGFP^−^. In RMCE2, the genomically integrated iRFP cassette was replaced with a Puro cassette and puromycin resistant cells were selected (Supplementary Figure S1, Step 3). Considering the possibility of simultaneously replacing multiple landing pads in one cell with the same cassette over two successive rounds of RMCE is extremely low, the obtained puromycin-resistant non-fluorescent clones (293F-Puro) should contain single landing pads on their genomes (this notion was later fully validated by genotyping of D1 clone, Supplementary Figure S2).

### T-RMCE improved integration efficiency in monoclonal cells carrying single landing pads

**FIGURE 3:**
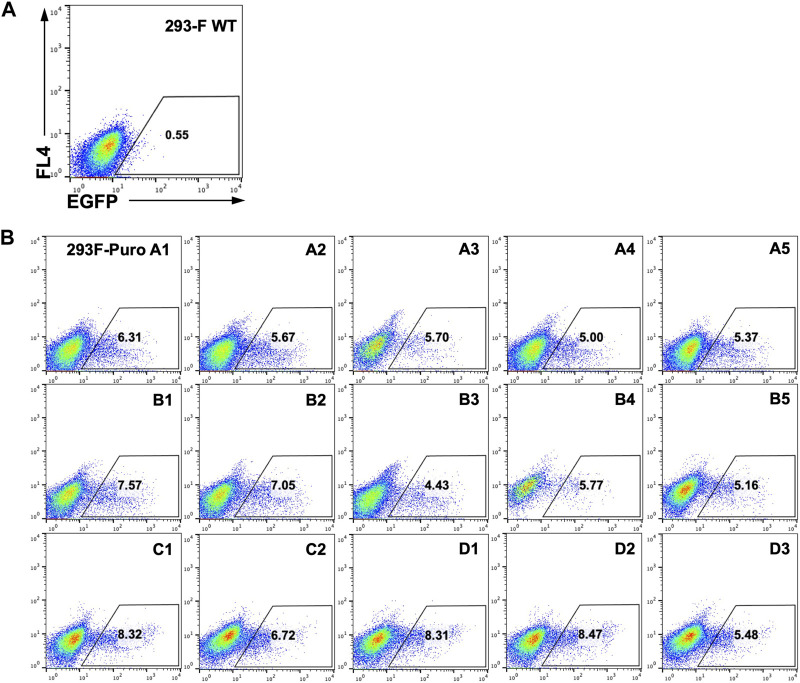
High efficiency of T-RMCE with monoclonal 293F-Puro cell lines. 293-F WT **(A)** and 15 RMCE-competent 293F-Puro clones **(B)** were subjected to T-RMCE (Figure 1B). GFP positive cells were detected by flow cytometry on day 3 post transfection.

As genomic loci of integrated landing pads can greatly impact SSR efficiency and GOI expression (Gaidukov et al., 2018), we next generated monoclonal RMCE-competent cell lines for T-RMCE tests. The single landing pad-bearing polyclonal 293F-Puro cells obtained above were subjected to serial dilution and monoclonal culture. Following puromycin selection, 15 cell clones were generated and flow cytometry analysis confirmed that all of them were iRFP^−^ and EGFP^−^. The obtained 293F-Puro cell clones were then subjected to T-RMCE with an EGFP donor cassette. Results indicated that T-RMCE efficiencies on day 3 post-transfection ranged from 4.4% to 8.5%, with an average of 6.4% and a standard deviation of 1.3% (Figure 3), significantly higher than the basal values of regular RMCE at ∼2% (Figure 2B). These results demonstrated that the improvement provided by T-RMCE applied to all tested clones. Particularly, clone D1 exhibited a high RMCE efficiency of 8.3% with intact cell morphology and viability, and thus was chosen for further studies.

### Landing pad genotyping of 293F-Puro D1 clone

To confirm landing pad monoclonality and determine its genomic locus, we genotyped the D1 clone by inverse PCR (iPCR) (Supplementary Figure S2A). Six restriction sites within the landing pad sequence–two at the upstream of Puro gene and four at its downstream–were chosen for iPCR. If a corresponding RE site is present nearby in the genome on the opposite stream of Puro gene (pseudo cutting sites shown as dashed lines in Supplementary Figure S2A), following single RE digestion and self-ligation, a circular DNA encoding Puro and its surrounding gDNA will be generated. Using primers specifically annealing to the Puro gene in the outward directions, the genomic DNA flanking the landing pad can be amplified and sequenced. Results of iPCR showed that dominant single bands of 4.8 kb, 0.9 kb, 2.3 kb, and 4.2 kb were obtained when the digestions were done with *Xho*I, *Psp*OMI, *Pvu*II, and *Afl*III respectively, a clear indication that a single landing pad was integrated into the D1 genome (Supplementary Figure S2B). Sanger sequencing of the iPCR products derived from three downstream RE digestions all pointed that the upstream of the landing pad was integrated to chromosome 6 within intron 6 of *PAKN* gene (Supplementary Figure S2C). Similarly, genotyping using the upstream RE *Afl*III revealed that the downstream of the landing pad was also integrated into *PAKN* intron 6. Notably, the faint bands generated by *Xho*I iPCR were also examined, but results showed no consecutive sequences connecting landing pad and chromosome regions, suggesting they were non-specific amplicons. Collectively, iPCR confirmed that D1 clone carries a single landing pad integrated at intron 6 of *PAKN* on Chr6.

### TAg expression increased the donor plasmid copy number

**FIGURE 4:**
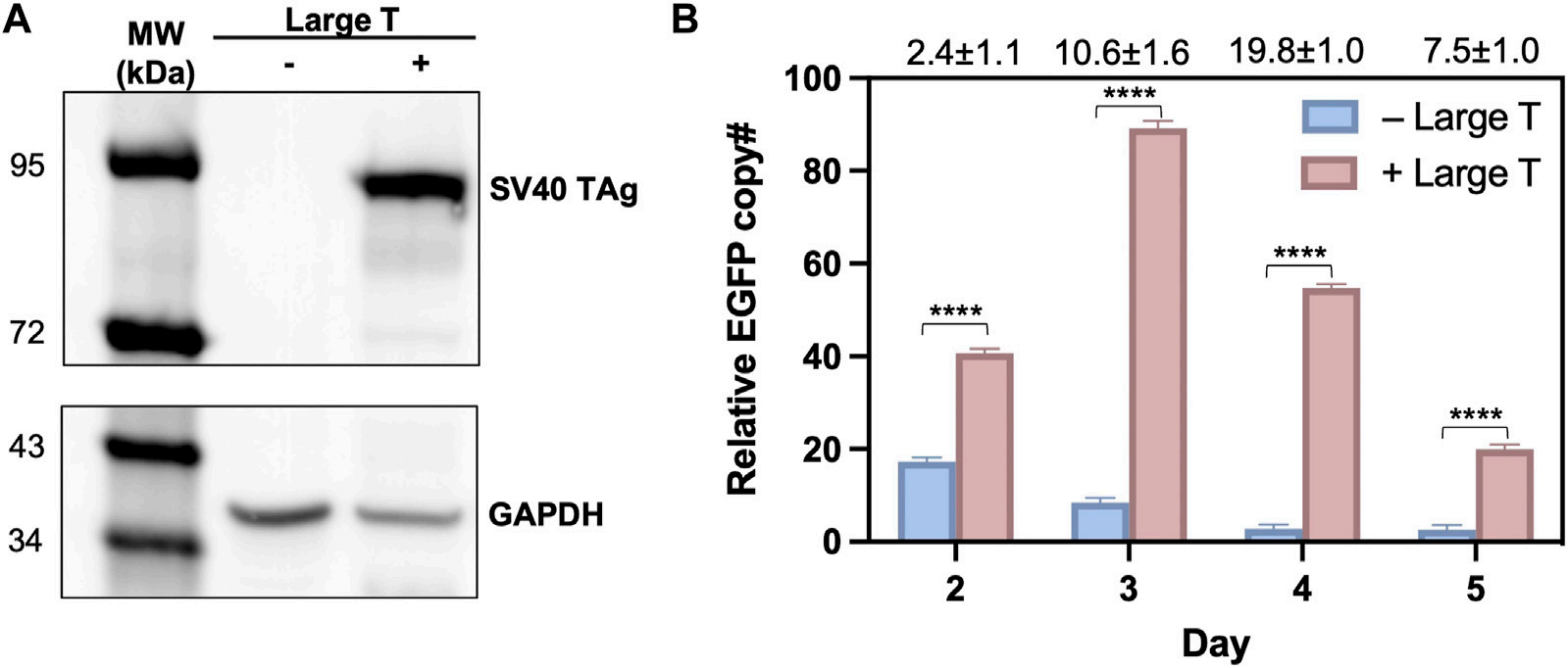
Large T expression profiles and quantification of donor plasmids in 293F-Puro D1 clone. **(A)** Western blot analysis of large T expression in whole cell samples transfected with/without pLargeT. GADPH was used as the loading control. **(B)** The relative copy number for EGFP, normalized with actin. 293F-Puro D1 cells were co-transfected with donor plasmid pSV-EGFP and pLargeT (+ Large T) or empty plasmid pcDNA (- Large T) without FLP/Cre. Total DNA was extracted on day 2–5 after transfection and subjected to qPCR quantifications (Supplementary Figure S1, *n* = 3). Folds of EGFP copy numbers with over without large T are shown on top. Error = S.D.; *p*-value was determined by one-tailed t-test (****, *p* < 0.0001).

To validate that the observed high efficiency of T-RMCE was correlated with donor DNA amplification, we measured the cellular amounts of EGFP donor plasmid by quantitative PCR. 293F-Puro D1 cells were co-transfected with the SV40 origin bearing donor plasmid and TAg expression plasmid, or its empty vector lacking TAg gene as the control. Notably, the recombinase expression plasmid was not added to avoid interference given by genomic integration. As expected, western blotting detected TAg expression only in the cells transfected with TAg gene (Figure 4A). On day 2–5 post transfection, total DNA samples were extracted for real-time PCR to determine the relative copy numbers (RCN) of EGFP gene normalized to actin (Supplementary Figure S3). Results showed that without TAg expression, the level of EGFP gene quickly decreased over time from 17.3 ± 0.9 on day 2 to 8.5 ± 1.0 on day 3 and 2.8 ± 0.9 on day 4 (all in RCN over actin), demonstrating a rapid diminishment of the donor plasmid (Figure 4B). In contrast, with TAg expression, the amounts of EGFP donor plasmid not only sustained but also increased substantially. Particularly, EGFP had RCN of 89.2 ± 1.6 on day 3 and 54.7 ± 0.9 on day 4, equivalent to a 10.6- and 19.8-fold donor DNA supply compared to that without TAg. Overall, these results clearly indicated that TAg expression mediated donor DNA replication in the transfected cells.

### Highest T-RMCE efficiency achieved on day 4 post transfection

**FIGURE 5:**
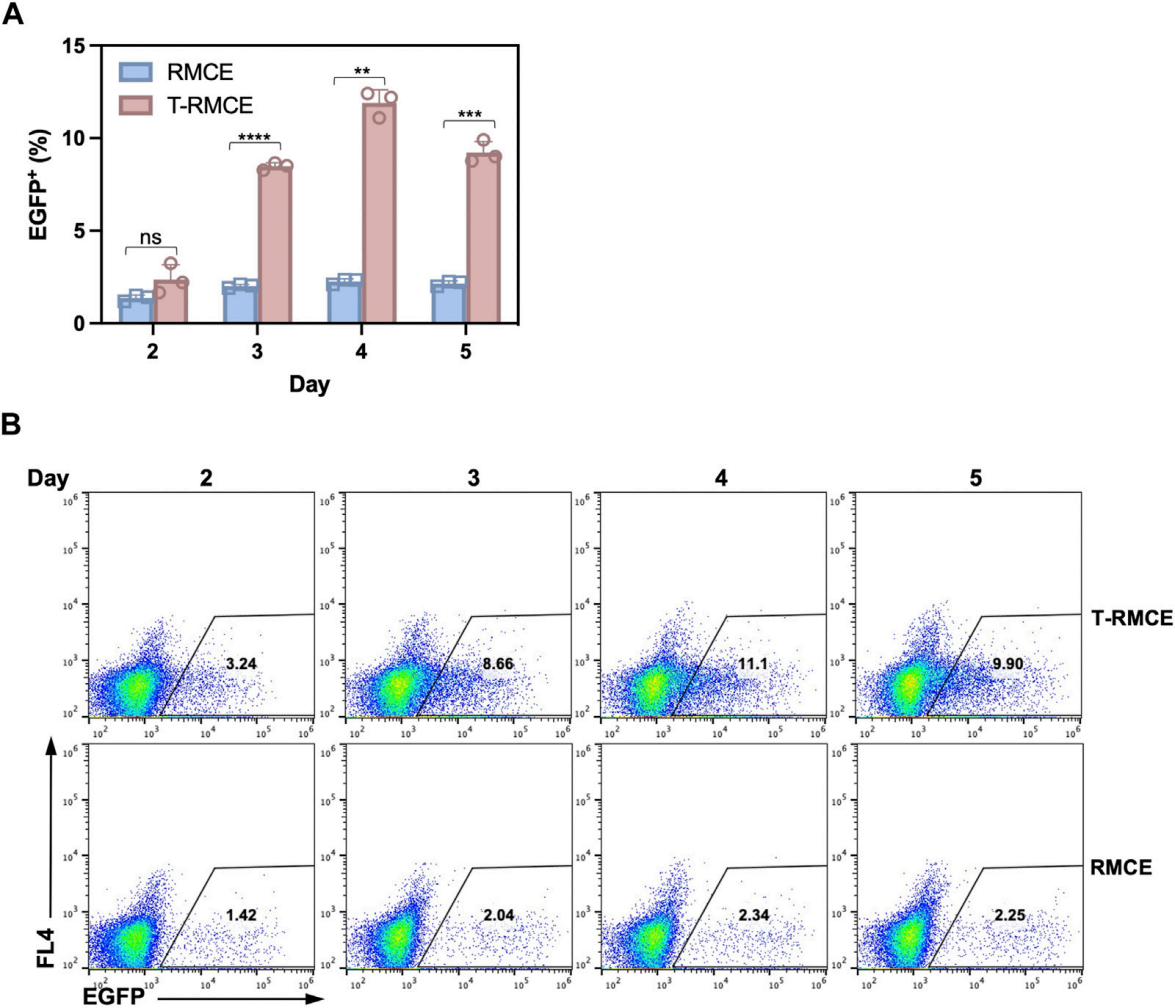
Effect of post-transfection cultivation time on T-RMCE efficiency. **(A)** Monoclonal 293F-Puro D1 cells were co-transfected with pSV-EGFP, pLargeT and pF2AC, and analyzed on 2–5 days after transfection. Empty plasmid pcDNA was used in replacement of pLargeT in control experiments. (*n* = 3). Error = S.D.; *p*-value was determined by one-tailed t-test (ns, not significant; *, *p* < 0.1; **, *p* < 0.01; ***, *p* < 0.001; ****, *p* < 0.0001). **(B)** Representative flow cytometry results.

The above observation that donor DNA amounts peaked on day 3 and maintained at high levels over several days (Figure 4B) encouraged us to test T-RMCE efficiency over the duration of day 2–5 post-transfection. Results of flow cytometry analysis showed that the percentage of EGFP^+^ cells were increased from 2.4% to 0.8% on day 2 to 8.5% ± 0.2% on day 3, maximized at 11.9% ± 0.7% on day 4, and slightly reduced to 9.2% ± 0.6% on day 5 (Figure 5). In contrast, without TAg, the RMCE efficiency slightly increased from day 2 at 1.4% ± 0.2% and plateaued at 2.3% ± 0.1% after day 3. The correlation between donor DNA copy number and integration efficiency clearly indicated that the availability of GOI was an important factor in RMCE—increasing cellular amounts of donor DNA significantly improved the RMCE efficiency. For T-RMCE, we also found a delay between the peak time for donor DNA copy number and that for the EGFP^+^ population, presumably due to the time required for RMCE integration and transgene expression. Collectively, the dynamics of T-RMCE process (Figure 4B; Figure 5A) provided strong evidence that TAg-mediated donor plasmid amplification indeed facilitated the genomic integration of RMCE. These results also guided us to harvest cells on day 4 post-transfection for the following work on library construction.

### Application of T-RMCE constructed libraries on GFP engineering

**FIGURE 6:**
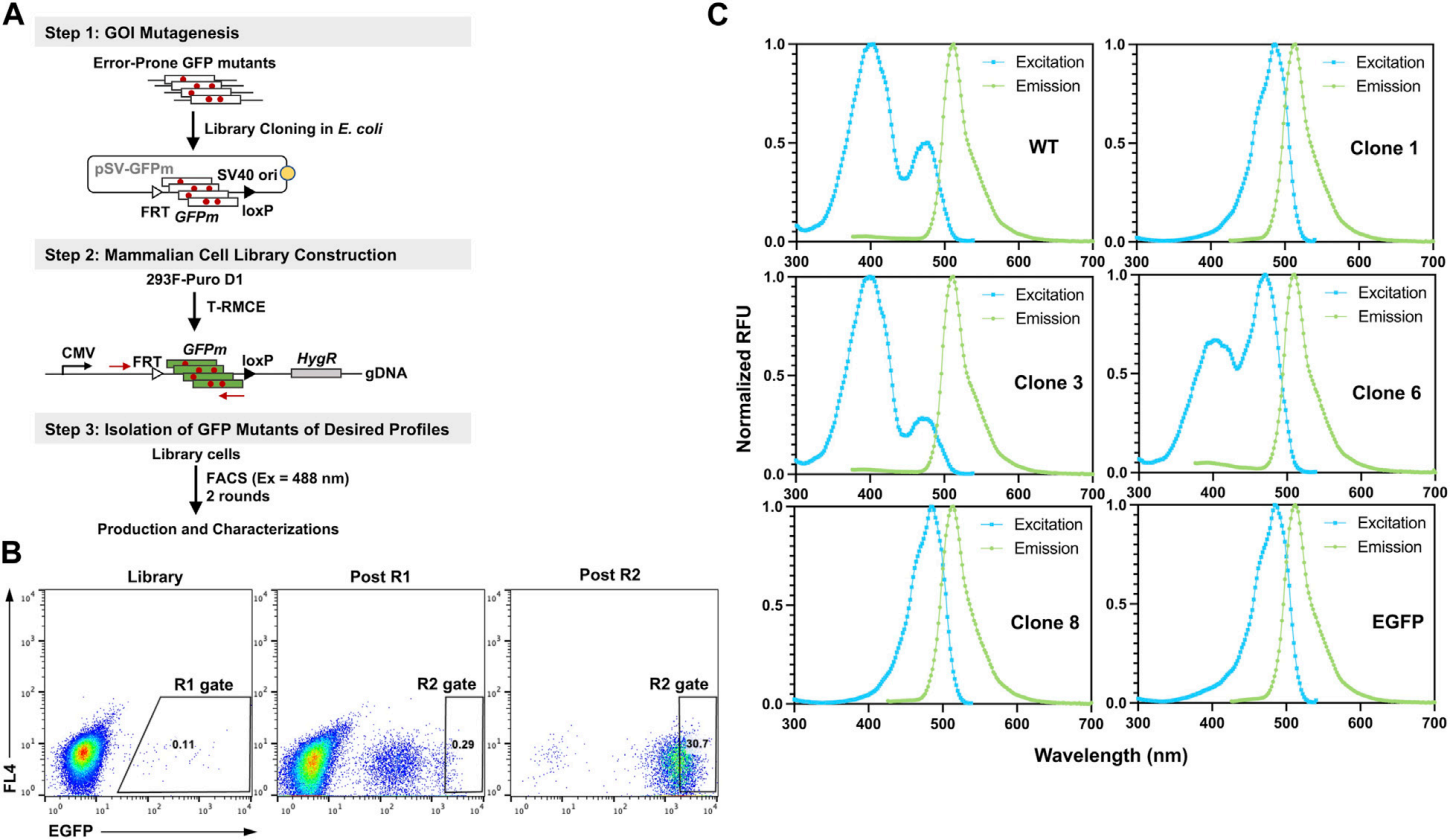
Engineering GFP with enhanced fluorescence under 488 nm excitation. **(A)** GFP library construction in mammalian cell via T-RMCE. (Step 1) Mutagenesis was introduced by error-prone PCR and library plasmids were cloned into *E. coli*. (Step 2) GFP mutant library construction in 293F-Puro D1 cells by T-RMCE. PCR primers for identification of GFP variants are shown. (Step 3) Isolation of GFP mutants by two rounds of FACS with 488 nm excitation and 526/48 emission. **(B)** Flow cytometry analysis of library, post round 1 (R1), and post round 2 (R2) cell populations. During R1 sorting, enrich mode was used and top 0.11% cells were collected. In R2, purity mode was used, and gate was set at 0.29%. **(C)** Excitation and emission spectra of isolated GFP variants. GFP wt and EGFP were used as control.

To demonstrate the utility of T-RMCE platform, we conducted directed evolution of GFP for improved fluorescence properties (Figure 6A). Random mutations in wild type GFP (80Arg) gene were introduced by error-prone PCR and cloning the mutated GFP fragments (GFPm) into the RMCE donor plasmid resulted in 1.08×10^8^ *E. coli* transformants. The obtained library plasmids, carrying the promoter-less FRT-GFPm-loxP cassette, were used to transfect 1×10^8^ 293F-Puro D1 cells. To ensure the high efficiency of T-RMCE applied to library construction, all parameters, including amounts of DNA, transfection agents and culture media, and flask usage, were scaled proportionally according to the cell number. Based on the observed RMCE efficiency of 12% transfected cells on day 4 (Figure 5A), presumably >1×10^7^ library diversity was obtained. With excitation at 488 nm and emission detected at 526/48 nm, two consecutive rounds of FACS were conducted. 2×10^8^ library cells were sorted in each round and the top 0.11% and 0.29% GFP^+^ cells were isolated using enrich mode in round 1 (R1) and purity mode in round 2 (R2), respectively (Figure 6B). Flow cytometry analysis of the library, post R1, and post R2 samples exhibited a significant shift of cell populations towards higher fluorescence intensities, a clear sign of successful enrichment of GFP variants with improved fluorescence properties (Figure 6B).

From post R2 cells, genomic DNA was extracted and the genes of isolated GFP variants were recovered and subcloned into *E. coli* for identification, production, and characterizations. Out of 16 randomly picked colonies, sequencing results identified four unique clones (Table 1). Among them, clone 1 was the most abundant (10/16) carrying a single mutation of Ser to Thr at position 65 (S65T), clone 3 had a mutation of F64L, clone 6 had a mutation of I167V, and clone 8 had T62S/S65T/P192L triple mutations. Recombinant production in *E. coli* indicated that for all variants except clone 6, decent yields were achieved at 87–103 mg purified proteins per liter of culture (Supplementary Figure S4; Table 1). While there was no notable difference on the emission spectra of isolated variants compared to that of GFP wt, the dominant excitation peak shifted from 395 nm of GFP wt to 488 nm of clone 6, and clones 1 and 8 exhibited a single excitation peak at 488 nm (Figure 6C). These results were expected as FACS was performed at the exact excitation wavelength. Molecular extinction coefficients and quantum yields of isolated variants were further measured by using GFP wt and EGFP as the references (Table 1; Supplementary Figure S5). Results showed that clone 1 and clone 3 exhibited improved fluorescence properties with their brightness intensities at 30,700 and 22,000 M^-1^ cm^-1^ respectively, representing 1.90- and 1.36-fold enhancements over GFP wt. Intriguingly, the S65T and F64L mutations identified in this study by global random mutagenesis and FACS in 293F cells were the key mutations of well-established EGFP discovered by targeted mutagenesis (Cormack et al., 1996). Our results, consistent with other studies, suggested that the S65T mutation was essential for suppressing its excitation at 395 nm (Heim et al., 1995), and F64L mutation was presumably responsible for its improved yield at 37°C (Arpino et al., 2012). Overall, we demonstrated that T-RMCE was amenable to construct large libraries, from which GFP variants with enhanced fluorescence were isolated.

**TABLE 1:** Characterizations of isolated GFP mutants.

Clone	Occurrence	Mutation	Yield (mg/L)	Ex (nm)	Em (nm)	Extinction coefficient (ε, M^-1^ cm^-1^)	Quantum yield	Brightness intensity (εQY, M^-1^ cm^-1^)	Reference
WT (80R)	-	-	80	395	512	21,000	0.77	16,170	Heim and Tsien (1996)
1 (80R)	10/16	S65T	103	488	512	59,940	0.51	30,700	This study
3 (80R)	3/16	F64L	93	394	512	16,640	0.74	12,290	This study
6 (80R)	2/16	I167V	13	488	512	n.d	n.d	n.d	This study
8 (80R)	1/16	T62S, S65T, P192L	87	488	512	54,570	0.40	22,000	This study
EGFP (80Q)	-	F64L, S65T, H231L	131	488	512	56,000	0.60	33,600	Cranfill et al. (2016)

n.d. = not determined.

## Discussion

### Novelty of T-RMCE

Site-specific recombination holds great promise for precise genomic modifications, but construction of monoclonal libraries with >10^7^ diversity often presents as a challenge. To improve RMCE efficiency, numerous methods developed so far mainly focused on engineering recombinase and their associated components (Buchholz et al., 1998; Bolusani et al., 2006; Anderson et al., 2012; Voziyanova et al., 2013; Shah et al., 2015; Voziyanova et al., 2016) or enhancing donor DNA transportation (Graessmann et al., 1989; Hebert, 2003; Escriou et al., 2003; Ondrej et al., 2007; Badding et al., 2012). Apart from all these reported approaches, this study develops a simple yet robust method to improve RMCE efficiency. The novelty of T-RMCE is based on *in situ* replication of donor DNA in nucleus. Our data indicated that TAg co-transfection mediated prolonged duration and up to 20-fold increases of the copy numbers of the SV40 origin bearing donor plasmid (Figure 4B). As a result, T-RMCE led to a dramatic enhancement of transgene integration efficiency, reaching 12% of transfected cells without antibiotic selection (Figure 5A). In contrast, in the absence of TAg, the cellular copy numbers of donor DNA quickly waned since day 2 of transfection and RMCE efficiency only plateaued at ∼2%. Notably, the time course profiles of donor DNA amount and RMCE efficiency clearly suggested their correlation, with 1 day delay observed between their peak times, presumably reflecting the period needed for recombination occurrence and GOI expression.

### Limitations of current study

It has been speculated that TAg residues 47 to 56 (PKKKRKVEDP) may encode a nuclear localization signal (NLS) peptide (Kalderon et al., 1984), which can facilitate the donor DNA translation across nuclear membrane, considering TAg’s ability to recognize the SV40 origin located on the plasmid. However, to distinguish the contributions of TAg-mediated donor DNA translocation and replication to SSR efficiency improvement, further study of using truncated TAg designs will be needed. As a DNA tumor virus, SV40 infects non-dividing cells, drives them into S-phase, and induces cell transformation (Ahuja et al., 2005). Mounting evidence suggests that these tumorigenic processes are mainly mediated via cooperation of its large and small T antigens, *e.g.*, to inhibit tumor suppressors p53, Rb, and pp2A (Porrás et al., 1999). In this study, as only TAg was introduced for transient expression, no significant viability or morphology issues were detected. However, the full impact of TAg expression on transfected cells warrants a complete investigation. Inverse PCR is a straightforward and effective approach for genotyping. Our analysis of 293F-Puro D1 clone clearly indicated a single landing pad inserted at intron six of *PRKN* gene (Figure 5). Interestingly, with its size over 1.4 Mb, *PRKN* is one of the largest human gene carrying huge introns. However, it is not clear whether this locus contributes to the high T-RMCE efficiency observed in this study, and thus further study will be needed.

### Suggestions on T-RMCE optimizations

The landing pad can be introduced to well-documented genomic safe harbors (GSH), *e.g.*, by CRISPR, to guarantee high expression and transgene stability (Papapetrou and Schambach, 2016; Hamaker and Lee, 2018). To reduce the transgene plasmid size, the unnecessary elements including f1 ori and hygromycin resistance marker were removed from the TAg and FLP-2A-Cre expression and donor DNA plasmids in this study. Designs based on minicircle DNA can be applied to further minimize the plasmid size and likely improve the transfection efficiency (Kay et al., 2010). Other optimizations may include using highly active SSRs (Durrant et al., 2023) or adjusting the ratio among the three transfected plasmids. In addition, construction of 293F cell lines stably expressing TAg could also be considered as well, as it allows to transfect more donor plasmid.

### Significance of T-RMCE

We developed T-RMCE with 293F cells, as its suspension feature gives great scale-up potential. Assuming the observed efficiency at ∼12% (Figure 5), T-RMCE of 2×10^9^ 293F suspension cell in 1 L media can create libraries of >2×10^8^ diversity, capable for most combinatory tasks. It is worth mentioning that other cell types, including CHO and Jurkat can also be the candidates for T-RMCE. And its design principle should be applicable to other SSR and nucleases as well. We demonstrated the utility of T-RMCE with GFP engineering. However, performing directed evolution in mammalian cells is highly valuable in cases where target proteins need mammalian machineries for proper post-translational modifications (*e.g.*, antibody Fc domain) and cellular functions (*e.g.*, T cell receptor). Overall, with advantages such as monoclonality, high fidelity, large cargo gene capacity, and importantly, efficiency improved by this work, we expect the developed T-RMCE can have broad applications in directed evolution and large-scale genomic screening.

## Material and methods

### Plasmid construction

*Landing pad plasmids:* DNA fragment encoding PGK promoter was chemically synthesized (IDT) and cloned into pcDNA3.1/Hygro^(+)^ at NsiI and XmaI sites to replace its SV40 promoter and SV40 ori with PGK promoter. The obtained pcDNA3.1/PGK-Hygro was digested with HindIII and XhoI and cloned with a synthesized DNA fragment encoding FRT-EGFP-loxP, to give ppHyFGL. To reduce plasmid size, pcDNA3.1^(+)^ was digested with PvuII, and the obtained 3.3 kb fragment was gel-purified and self-ligated to give pcDNA. Fragments encoding PGK promoter and FRT-Puro-loxP were fused by overlapping PCR then inserted into pcDNA at BglII and BamHI sites to give ppPuro. *Donor plasmids:* The 136 bp SV40 origin DNA was amplified using pcDNA3.1^(+)^ as the template and cloned into pcDNA using BglII and BamHI to give pcDNA-SV40ori, in which the CMV promoter was removed. Fragment encoding FRT-iRFP-LoxP was chemically synthesized and cloned into pcDNA-SV40ori at BglII site resulted in pSV-iRFP. pSV-Puro and pSV-EGFP were prepared similarly. *Recombinase plasmid:* DNA fragment encoding FLP (P2S, L33S, F70L, Y108N, S294P) (O'Gorman et al., 1991; Buchholz et al., 1998) and Cre (Buchholz et al., 1996) separated by a T2A self-cleaving peptide (F2AC (Anderson et al., 2012)) was assembled by overlap PCR. The pcDNA was modified by insertions of a chimeric intron (Gentsch et al., 2007) and a Woodchuck hepatitis virus posttranscriptional regulatory element (WPRE) (Pañeda et al., 2006) at NheI/KpnI and XhoI/XbaI sites respectively. Obtained pCIW was digested with KpnI/XhoI and ligated with F2AC to generate pF2AC. *Large T plasmid:* SV40 TAg gene (Gene ID 29031019) was amplified by PCR using the genomic DNA of HEK293T as template and cloned into pcDNA at NheI and XbaI sites to give pLargeT. Restriction enzymes, polymerases and DNA ligase were purchased from NEB. All cloned plasmids were confirmed by sequencing.

### Cell line and cell culture

Expi293F cells (Thermo Fisher Scientific) were maintained in Expi293 expression media (Gibco, Cat#A1435101) supplemented with 40 U/mL penicillin, and 40 μg/mL streptomycin (Gibco, Cat#15140122) in 125 mL flasks at 37°C under 8% CO_2_ in humidified atmosphere with orbital shaking at 130 rpm.

### Generation of RMCE competent cell lines

To generate RMCE ready 293F-puro or 293F-EGFP polyclonal cells, 2×10^7^ Expi293F cells were transfected with 10 µg ppPuro or ppHyGFL and 30 µg PEI MAX 40K (Polysciences, Cat#24765–1) in 10 mL Expi293 media followed by cultivation for 3 days; cells were then selected in fresh media supplemented with the corresponding antibiotics for 7 days: ppPuro with 5 μg/mL puromycin (Gibco, Cat#A1113803) and ppHyGFL with 250 μg/mL hygromycin. To select cells with single landing pad, two rounds of consecutive RMCE were performed as previously described with modifications (Chen et al., 2016). Briefly, the obtained 293F-EGFP cells were co-transfected with pSV-iRFP and pF2AC and the iRFP^**+**^ and EGFP^**-**^ cells were sorted by FACS. After expansion, the 293F-iRFP cells were co-transfected with pSV-puro and pF2AC. On day 2 after transfection, cells were seeded into 96-well plates at 200 cells per well and selected in DMEM supplemented with 10% FBS and 5 μg/mL puromycin. After 7-day cultivation, 293F-Puro single cell clones were identified and expanded for further experiments.

### RMCE transfection

Cells were maintained in Expi293 expression media without antibiotics for 2 days, and 30 min before transfection, cells were seeded in fresh media at the density of 2×10^6^/mL. A mixture of 1 µg donor plasmid, 1 µg pF2AC and 6 µg PEI were prepared in 0.2 mL Expi293 media and added to 1.8 mL cell culture. For large T-RMCE, 4×10^6^ cells were transfected with a mixture of 6 µg PEI and 2 µg total plasmids including the donor plasmid, pLargeT, and pF2AC at a mass ratio of 1:1:2 or 1:1:1. Control transfections without pLargeT or pF2AC were carried out by replacing with same amounts of pcDNA3.1/Hygro^(+)^. For GFP library transfection, 1×10^8^ 293F-Puro D1 cells were transfected with 25 µg pSV-GFPm, 25 µg pLargeT, 50 µg pF2AC and 400 µg PEI. 5×10^6^ 293F-Puro D1 cells were transfected similarly without pF2AC for qPCR and western blot analysis.

### Flow cytometry and fluorescence activated cell sorting

Cell analysis and sorting was performed on a S3e cell sorter (Bio-Rad), equipped with 488/640 nm dual lasers, and associated bandpass/longpass filters. EGFP/GFP mutants and iRFP were excited at 488 nm and 640 nm and detected with 526/48 nm filter (FL1 channel) and 700 nm/LP filter (FL4 channel), respectively. In general, 40,000 events were collected for analysis; FACS for GFP variants screening was performed in purity mode at 2000–3,000 events/s and the top 0.1%–0.3% of the GFP positive cells were collected.

### Genotyping of the landing pad genomic locus

Inverse PCR was employed to validate the single copy of landing pad and to determine its genomic locus in the 293F-Puro D1 cell line. Briefly, 15 ng genomic DNA was digested with one of the following restriction enzymes AflII, AgeI, BamHI, NheI, PspOMI, PvuII, or XhoI in a 10 µL reaction for 5 h followed by heat inactivation at 80°C for 20 min. The digestion mixture was then added with 40 µL of 1×T4 DNA ligase buffer containing 64 units of T4 DNA ligase and incubated at 16°C overnight. The self-ligated products were amplified using nested PCR. The second PCR products were examined by electrophoresis and extracted for Sanger sequencing.

### qPCR

After incubation with transfection mixtures for 24 h, 293F-Puro D1 cells were washed 3 times with 10 mL PBS to remove extracellular plasmids. Cells were seeded in 5 mL fresh media in a new flask and cultured for another 24 h. Then 1×10^6^ cells were collected, and the remaining cells were cultured in 5 mL fresh media. Cell sampling and passaging were repeated for three more days, to obtain cell samples of 2–5 days post transfection. The collected cells were washed with 1 mL PBS for 8 times and then underwent genomic DNA extraction with Wizard Genomic DNA Purification Kit (Promega Cat#A1120). 25 μL reaction mixtures were prepared in low profile 96-well PCR plates (Bio-Rad Cat#MLL9601) containing 100 ng template DNA, 100 nM each primer designed by Primer Premier 5 (Premier Biosoft), and 12.5 µL iQ5 SYBR Green SuperMix (Bio-Rad Cat#170–8,880). Reactions were proceeded on CFX Connect (Bio-Rad), and a final dissociation step was performed to obtain the melting curves (thermal profile) of the amplicons obtained in the reactions. Standard curves were generated using four of 10-fold serial dilutions of template DNA at day 1 post transfection with pSV-EGFP and pLargeT. Both water and the cells without transfection were used as controls, and actin was used for normalization. All qPCR experiments were performed in triplicates except duplicates for the standard curves. qPCR data was analyzed with Pfaffl method (Pfaffl 2001). Briefly, relative EGFP copy numbers were calculated with equation:

$$Ratio=\frac{{\left(E_{Actin}+1\right)}^{{Ct}_{Actin}}}{{\left(E_{EGFP}+1\right)}^{{Ct}_{EGFP}}}$$

where E is the qPCR efficiency derived from standard curves.

### Western blot

1×10^6^ 293F-Puro D1 cells transfected with pSV-EGFP and pLargeT or pcDNA were collected and washed with 1 mL PBS once, then resuspended in 100 µL 2 × reducing Lammeli buffer and boiled at 99°C for 10 min. After centrifugation at 17,000 *g* for 5 min, 10 µL of the supernatant sample was separated by 10% SDS-PAGE and transferred to a PVDF membrane. The membrane was blocked with 5% skim milk in PBS and detected with anti-SV40 T Ag antibody HRP (1:200, Santa Cruz Biotechnology Cat# sc-147 HRP). Chemiluminescence was developed by using SuperSignal substrate (Thermo Scientific Cat#34075). The amount of total protein was assessed by re-probing with anti-GAPDH-HRP antibody (1:3,000, Abcam Cat# ab105428).

### GFP library construction

Mutations were generated by error-prone PCR using the plasmid carrying a GFP clone Q80R gene as the template. The reaction mixture contained 10 mM Tris⋅HCl (pH 8.3), 50 mM KCl, 6.5 mM MgCl_2_, 1 mM dCTP, 1 mM dTTP, 0.2 mM dATP, 0.2 mM dGTP, 0.3 µM 5′primer, 0.3 µM 3′primer, 25.6 pM template DNA pSV-EGFP, 0.5 mM MnCl_2_, and 0.05 U/μL Taq DNA polymerase. The mutated GFP fragments were cloned into pSV-Puro at HindIII and XhoI sites by electroporation of *E. coli* SS320 competent cells. Library plasmid DNA was then miniprepped and ready for transfection to 293F-Puro D1 cells.

### GFP variants production and characterization

Post the second round of FACS, genomic DNA was extracted from 1×10^6^ cells using Wizard Genomic DNA Purification Kit. The fragments encoding GFP variants were PCR amplified with the primers recognizing the untranslated region on integrated landing pad at 5′and the C-terminal of GFP mutants at 3’. The PCR products were gel purified, digested with NheI and XhoI, and ligated into pET28a vector for Sanger sequencing to identify GFP mutants. GFP variants with a 6×His tag fused at their N-termini were expressed in *E. coli* BL21 (DE3) in Luria Bertani (LB) media containing 50 μg/mL kanamycin at 37°C. Overexpression was induced when OD_600_ reached 0.6 with addition of isopropyl-beta-D-thiogalactopyranoside (IPTG) to a final concentration of 1 mM. After incubation with shaking at room temperature for 12 h, the cells were harvested by centrifugation and sonicated at 4°C in lysis buffer (20 mM Tris pH 7.9, 500 mM NaCl, 20 mM imidazole). GFP variants were purified using HisPur Ni-NTA resin (Thermo Fisher Scientific #88222). Concentrations of purified fluorescent proteins were determined by spectrometry at 280 nm. The excitation spectra were scanned from 300 to 700 nm with emission at 560 nm, and absorbance was measured at the excitation maximum wavelength (Ex). The emission spectra were scanned from 300 nm to 700 nm with excitation at Ex (430 nm for EGFP, variants one and 8; 370 nm for wild type (WT) GFP and variant 3), and the integrated fluorescence intensity was calculated from 460 to 670 in GraphPad Prism 9. The extinction coefficient and quantum yield were measured as described previously (Cranfill et al., 2016). For each fluorescent protein, 30–100 mg/L five concentrations were tested in 20 mM Tris pH 7.9, 500 mM NaCl. WT GFP was used as standard for variants 3, and EGFP was used as standard for variants one and 8. A blank was measured and subtracted from all the spectra. All measurements were performed using 96-well optical-bottom plate (Thermo Scientific Cat#165306) on BioTek Cytation 5. Plots of the absorbance as a function of the concentration were generated with the slopes as M and plots of the integrated fluorescence intensity as a function of absorbance were generated with the slopes as N. The extinction coefficient ε and the quantum yield QY were calculated using the equations below:

$$\epsilon_{sample}=\frac{\epsilon_{standard}\times M_{sample}}{M_{standard}}$$

$${QY}_{sample}=\frac{{QY}_{standard}\times N_{sample}}{N_{standard}}$$

Brightness=∈×QY

## Data Availability

The original contributions presented in the study are included in the article/Supplementary Material, further inquiries can be directed to the corresponding author.
