# Supplementary material for: Large T antigen mediated target gene replication improves site-specific recombination efficiency
Source: Front Bioeng Biotechnol. 2024 Apr 26;12:1377167. doi: 10.3389/fbioe.2024.1377167 (PMC11082406; doi:10.3389/fbioe.2024.1377167)
Supplement: Supplementary file 1 [file DataSheet1.docx]

Supplementary Information

Large T antigen mediated target gene replication improves site-specific recombination efficiency

Zening Wang^1,2^, Chuan Chen^2^, Xin Ge^1,2,^*

^1^Institute of Molecular Medicine, University of Texas Health Science Center at Houston, Houston TX 77030, USA.

^2^Department of Chemical and Environmental Engineering, University of California Riverside, Riverside CA 92521, USA.

*Corresponding author:

E-mail:  [xin.ge@uth.tmc.edu](mailto:xin.ge@uth.tmc.edu)

**Content:**

**Figure S1.** Generation of RMCE-competent 293F monoclonal cells carrying a single landing pad.

**Figure S2.** Genotyping of 293F-Puro D1 clone.

**Figure S3.** EGFP gene quantification by qPCR.

**Figure S4.** SDS-PAGE of purified GFP variants.

**Figure S5.** Characterizations of GFP variants.

**Figure S1:**

**

**

**Figure S1. Generation of RMCE-competent 293F monoclonal cells carrying a single landing pad.** (Step 1) EGFP expression cassette is randomly integrated into genomic loci under hygromycin selection to give EGFP positive cells (293F-EGFP). (Step 2) In RMCE1, the *EGFP* gene at the landing pad is replaced with an *iRFP* gene on the donor plasmid pSV-iRFP, and iRFP^+^ EGFP^-^ cells are selected by FACS to give 293F-iRFP. (Step 3) In RMCE2, the *iRFP* gene at the landing pad is replaced with a puromycin resistance gene on the donor plasmid pSV-Puro to give 293F-Puro. Due to low occurrence of multiple replacements, two successive rounds of RMCE guaranteed that resultant 293F-Puro cells carry a single expression RMCE landing pad. Obtained 293F-Puro cells were then subjected to monoclonal selection. Notably, the uncolored rectangles on the promoter-less donor plasmids indicate no expression, while the coloured rectangles on gDNA indicate expression enabled after integration at downstream of the CMV promotor. Right panels show flow cytometry results for each step.

**Figure S2:**

**
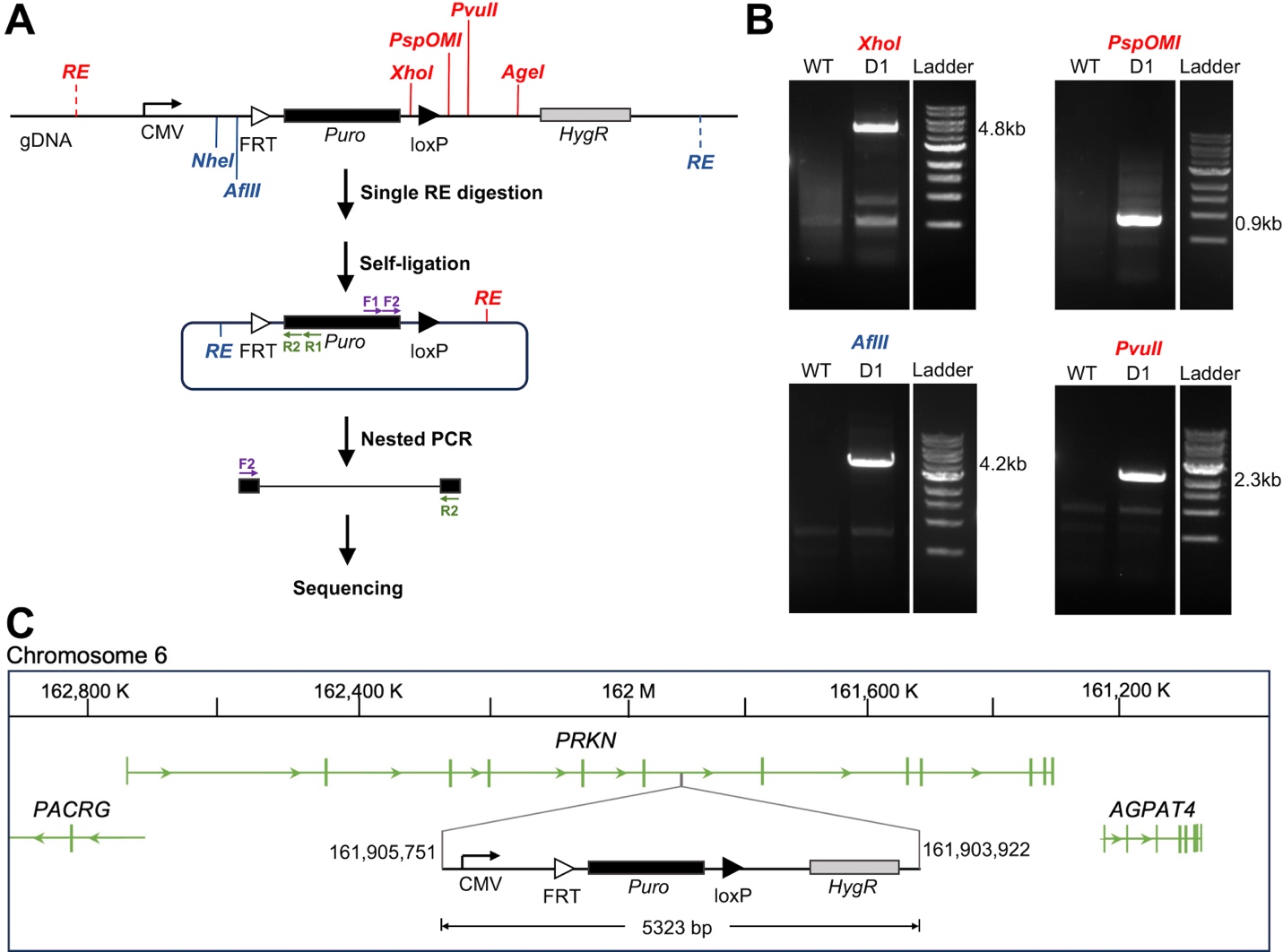
**

**Figure S2. Genotyping of 293F-Puro D1 clone. (A)** Schematic of inverse PCR (iPCR) for landing pad genotyping. Extracted genomic DNA was digested with one of indicated restriction enzymes, self-ligated, and subjected to iPCRs in which primers were designed to outwardly anneal at the integrated puromycin resistance gene region. FRT and LoxP recognition sites are shown as triangles. **(B)** Electrophoresis analysis of iPCR products with four REs. **(C)** Genomic context of 293F-RMCE D1 landing pad revealed by Sanger sequencing. Human genome sequence (GRCh38.p14) was used as the reference.

**Figure S3:**


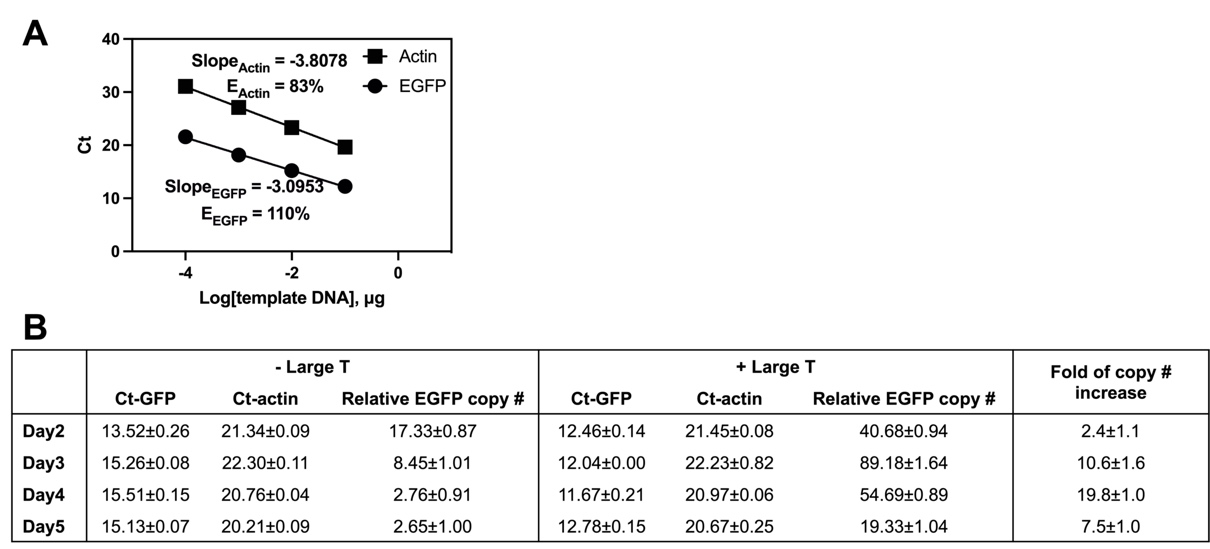


**Figure S3. EGFP gene quantification by qPCR. (A)** Calibration curves for EGFP and actin. The qPCR efficiency E was calculated as (10^-slope^-1) × 100%. **(B)** Cycle threshold (Ct) values of qPCR. The relative copy number for EGFP, normalized with that of actin, was calculated as $\frac{{{(E}_{Actin}+1)}^{{Ct}_{Actin}}}{{{(E}_{EGFP}+1)}^{{Ct}_{EGFP}}}$ . Fold of copy number increase was calculated as the ratio of relative EGFP copy number with large T over that without large T (*n* = 3).

**Figure S4:**

**
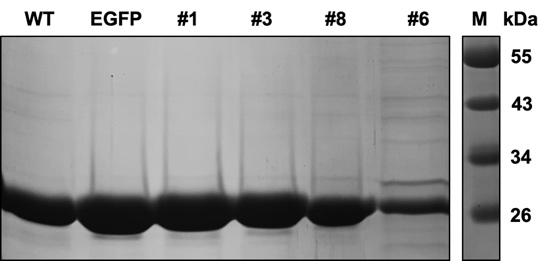
**

**Figure S4. SDS-PAGE of purified GFP variants.**

**Figure S5:**

**
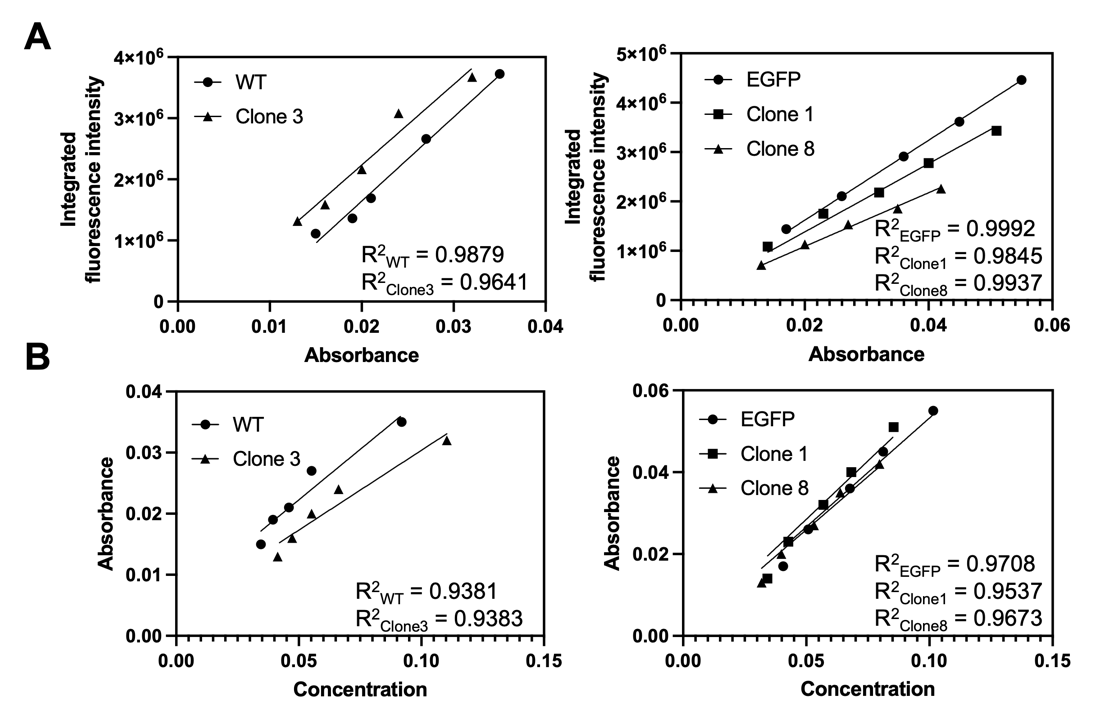
**

**Figure S5. Characterizations of GFP variants.** Linear plots for **(A)** fluorescence quantum yield and **(B)** extinction coefficient calculation. R^2^ values are shown.
